# Complete response to ipilimumab and nivolumab therapy in a patient with extensive extrapulmonary high-grade small cell carcinoma of the pancreas and HIV infection

**DOI:** 10.1186/s40425-018-0379-x

**Published:** 2018-07-09

**Authors:** Muhammad Husnain, Wungki Park, Juan Carlos Ramos, Thomas E. Johnson, Joseph Chan, Arvind Dasari, Raja Mudad, Peter J. Hosein

**Affiliations:** 1 Department of Medicine, Division of Oncology, University of Miami Miller School of Medicine and Sylvester Comprehensive Cancer Center, 1475 NW 12th Ave. Suite 3400, Miami, FL 33136 USA; 2 Department of Ophthalmology, University of Miami Miller School of Medicine and Bascom Palmer Eye Institute, Miami, USA; 3 Department of Medicine, Division of Infectious Diseases, University of Miami Miller School of Medicine, Mount Sinai Medical Center, Miami, USA; 4 MD Anderson Cancer Center, Houston, USA

**Keywords:** Check point inhibitors, Immunotherapy, HIV, Small cell cancer, Neuroendocrine carcinoma

## Abstract

**Background:**

Immune checkpoint inhibitors (CPIs) have shown promising results in many solid tumors. There are limited data on the safety and efficacy of these drugs in HIV infected patients as they have traditionally been excluded from CPIs clinical trials.

**Case presentation:**

We present a case of an HIV-positive patient with extensive extrapulmonary high-grade small cell carcinoma who was treated with dual CPIs (nivolumab and ipilimumab) with a complete response to therapy and with a manageable safety profile. We performed a comprehensive literature review identifying 62 total HIV positive cases treated with CPIs showing this to be a potentially safe option in HIV-positive patients.

**Conclusion:**

HIV infection is not an absolute contraindication to CPI therapy. Our case and others provide justification for ongoing trials of CPI therapy in patients with HIV infection, a group that has traditionally been excluded from clinical trials.

## Background

Immune checkpoint inhibitors (CPIs) including anti-programmed cell death 1 (PD-1), anti-PD ligand 1 (PD-L1) and anti-cytotoxic lymphocyte associated protein 4 (CTLA-4) antibodies have shown promising results in several solid cancers including small cell lung cancers. Small cell lung cancer (SCLC) is a poorly differentiated high-grade neuroendocrine carcinoma representing 15% of all lung cancers with very poor prognosis [1]. CheckMate-032 and KEYNOTE-028 showed modest activity of CPIs in recurrent SCLC patients and high-grade pancreatic neuroendocrine carcinomas [2, 3]. CheckMate-032 was a phase I/II trial of nivolumab (anti-PD-1 antibody) alone or nivolumab plus ipilimumab (anti-CTLA-4 antibody) in recurrent SCLC patients showing acceptable toxicity and clinically meaningful responses with 1-year overall survival rate of 27 and 48% for nivolumab alone and nivolumab plus ipilimumab arms respectively. KEYNOTE-028 studied the role of pembrolizumab (Anti-PDL1 antibody) in pancreatic neuroendocrine tumors and carcinoid tumors showing stable disease in 88 and 69% of patients respectively with a median follow up of 20 months. All the CPI trials excluded patients with chronic infections including HIV. There is limited data on the safety of CPI in HIV positive patients. We present a case of an extrapulmonary small cell cancer of pancreas in an HIV-positive patient who had a complete response to dual CPI therapy. We also review all the evidence available to date on the safety of check point inhibitors in HIV-positive patients.

## Case presentation

**Fig. 1:**
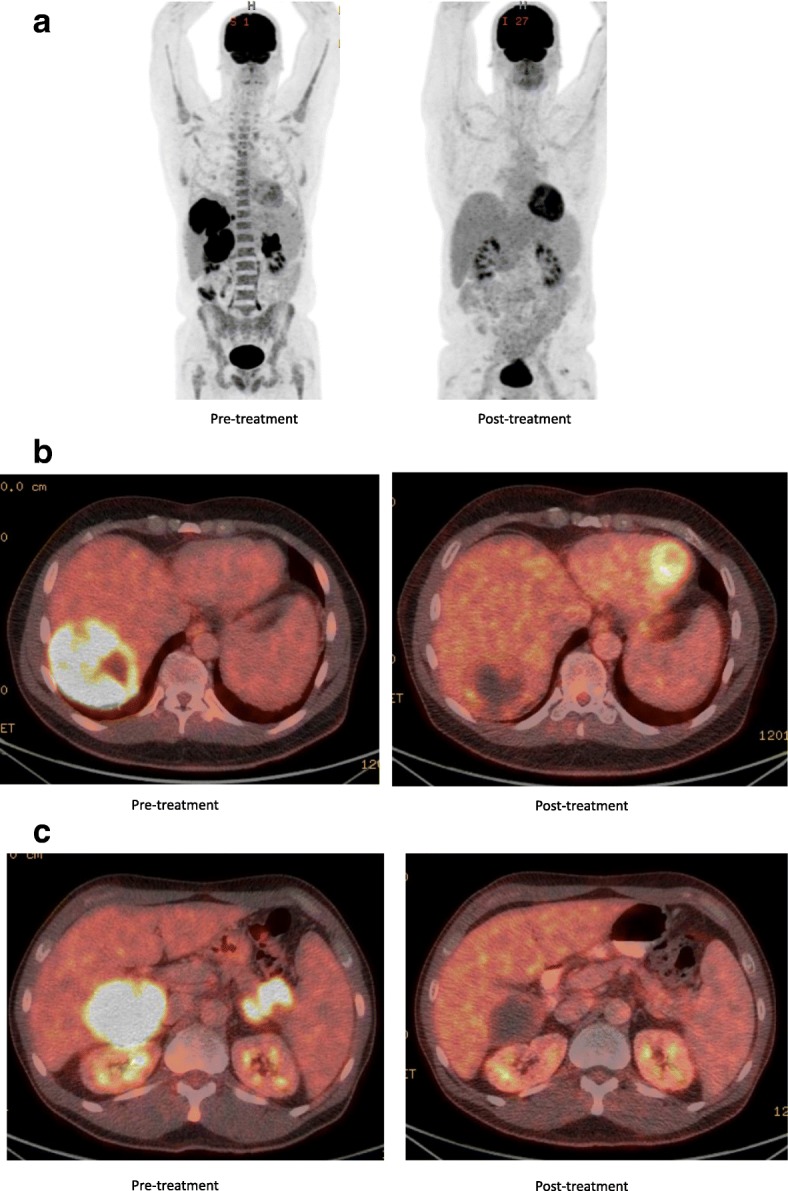
Positron emission tomography (PET) images **a**, **b** and **c**: Showing increased uptake in both liver and pancreatic lesions pretreatment with complete response post treatment

**Fig. 2:**
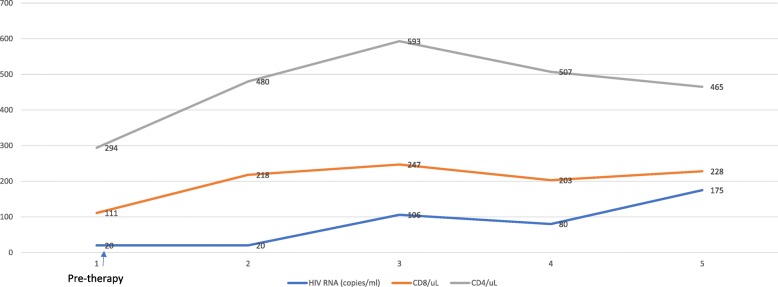
Line graph showing trends in HIV RNA, CD4 and CD8 cells (x axis showing time in months since the initiation of therapy)

A 52-year-old man was first diagnosed with HIV in 1991. He was maintained on antiretroviral therapy with emtricitabine-tenofovir and raltegravir. The HIV viral load was undetectable (less than 20 copies/ml) and the CD4 count of 850 cells/uL at the time of presentation. In December 2016, he presented to the emergency department with chief complaint of diplopia. A magnetic resonance imaging (MRI) of the orbits revealed a mass in the left orbit with involvement of the optic nerve. He was referred to ophthalmology and underwent a lateral orbitotomy and removal of the orbital mass. Pathology showed metastatic small cell carcinoma. A Computed Tomography (CT) scan of the chest, abdomen and pelvis and a Positron Emission Tomography (PET) scans were negative for any intrathoracic mass; however, there were multiple liver lesions and a large pancreatic tail mass. Given these findings his final diagnosis was extrapulmonary high-grade small cell carcinoma of the pancreas. Next Generation Sequencing of his tumor showed an intermediate tumor mutation burden with 9 mutations/megabases and deleterious alterations in *TP53, MLL3, MEN1, FAT1, CDKN2A, BCORL1, BCOR, ATRX* and *TSC2* genes. There is currently no approved targeted therapy for any of these mutations. He was started on chemotherapy with carboplatin and etoposide. He had a partial response (PR) after 2 cycles of chemotherapy. He had disease progression after 6 cycles of chemotherapy with carboplatin and etoposide. He was then started on chemotherapy with FOLFIRINOX (5-Fluorouracil, irinotecan, leucovorin and oxaliplatin) as second line therapy. He received four cycles but continued to have disease progression on imaging. He was then treated with carboplatin and paclitaxel but his disease continued to progress with clinical deterioration and significant abdominal pain. At that point, treatment with dual CPI therapy (nivolumab and ipilimumab) was pursued given the available data in refractory SCLC. Before the start of this therapy, his CD 4 count was 294 cells/uL with an undetectable HIV viral load (less than 20 copies/ml). He received Nivolumab 1 mg/Kg along with Ipilimumab 3 mg/kg every 3 weeks. After 2 doses of the combination, he developed acute kidney injury with creatinine of 4.2 mg/dl from a baseline of 1.0. The therapy was suspended and he was admitted to the hospital and a renal biopsy was performed which showed severe drug-induced acute interstitial nephritis (AIN). He was treated with high dose steroids; 500 mg of IV methylprednisolone for 3 days followed by a steroid taper. His renal function improved after 4 weeks with return of creatinine to baseline. He was then re-started on single agent Nivolumab at 1 mg/Kg and later ipilimumab at 1 mg/kg was added. The patient had significant clinical improvement soon after starting the dual CPI therapy with resolution of abdominal pain which previously required high-dose opioids. Repeat scans at 12 weeks (including MRI of the head and PET/CT scan) showed complete response (CR) as per PERCIST criteria with disappearance of all metabolically active lesions (Fig. 1). Patient was continued on antiretroviral therapy. The HIV viral load was undetectable before starting the CPIs (< 20 copies/ml) and increased to a high of 175 copies/ml. At the same time his absolute CD4 count increased from 294 cells/uL before treatment to a high of 593 cells/uL. His CD8 count also followed the similar pattern. It was 111 cell/uL before starting treatment and reached a high of 247 cells/uL (Fig. 2). The patient’s complete radiological response is still ongoing at the time of this report (24 weeks after the start of the dual CPI therapy).

## Discussion and conclusions

Many cases of exceptional and durable responses to CPIs have been reported. However, this case is unique in that it highlights the impressive efficacy of dual CPIs in a HIV positive patient. The only drug-related adverse effect was acute interstitial nephritis (AIN), successfully treated with high dose steroids. The patient achieved a complete clinical and PET response. Patient continued on antiviral therapy during the entire course of therapy. There was no significant adverse effect on the HIV viral load and CD4 cell counts. Interestingly, his viral load which was undetectable before the start of the CPI therapy increased to 106 copies/ml at week 10 followed by a drop in HIV viral load to 80 copies/ml at week 12. At the same time his CD4+ cell count increased to 593 cells/Ul at week 10 and dropping down to 507 cells/Ul at week12. Day et al. have shown that in untreated HIV infection PD-1 was significantly upregulated on HIV specific CD4 and CD8 T cells leading to T cell exhaustion and PD-1 blockade resulted in proliferation of HIV specific CD4 and CD 8 T cells and decrease in viral load [4, 5]. Guihot et al. treated an HIV positive patient with lung cancer with nivolumab showing a transient reactivation of HIV replication within CD4 T cells together with HIV specific CD8 T-cell activation that might be responsible for killing these reactivated HIV-producing cells resulting in an elimination of HIV reservoir and potential cure of HIV [6]. Smari et al. reported similar findings in an HIV positive patient with non-small cell lung cancer (NSCLC) who was treated with nivolumab and they concluded that nivolumab is successful at enhancing the HIV-specific CD8 T cells to proliferate and secrete cytokines, with promising results on the decrease of the HIV reservoirs [7]. In fact, a clinical trial of anti-PD-L1 antibody BMS-936559 in HIV positive patients is underway to determine the safety and efficacy of CPIs in HIV-positive patients as a therapeutic option to suppress HIV viral loads [8].

We performed a comprehensive literature review for use of CPIs in HIV positive patients. Search terms “immunotherapy”, “PD-1”, “CTLA-4”, “PD-L1”, “Checkpoint inhibitors”, “nivolumab”, “ipilimumab”, “pembrolizumab”, “HIV”, “AIDS” were used to search PubMed, congress abstracts from the annual meetings of the American Society of Clinical Oncology, European Cancer Congress, Society of Immunotherapy of cancer, American Association of Cancer Research and Google Scholar and for ongoing trials in ClinicalTrials.gov.

Our search identified 12 case reports [6, 9–18], 3 retrospective case series [7, 19, 20] and 1 prospective phase 1 clinical trial (NCT02595866) for HIV positive patients being treated with checkpoint inhibitors (Table 1). Uldrick et al. (NCT02595866) presented the interim analysis from Cancer Immunotherapy Trials Network-12 (CITN-12): A phase 1 study of pembrolizumab in patients with HIV and relapsed, refractory or disseminated malignancies. Seventeen HIV- positive patients with various malignancies were treated with pembrolizumab 200 mg intravenously every 3 weeks without any significant adverse effects on CD4+ cell count and HIV viral load. Rai et al.*....* reported a series of 44 patients with malignant tumors and concurrent solid organ transplant, HIV, HBV or HCV infection treated with CPIs. Among these, 11 were HIV-positive and there were no adverse effects on their HIV viral load and CD 4+ cell counts [20]. Similarly, Heppt et al and Samri et al treated 10 and 12 HIV positive patients respectively with CPIs showing similar results [7, 19]. Several other phase I/II clinical trials (NCT02408861 and NCT03304093) are underway to test the safety and efficacy of CPIs either alone or in combination in patients with HIV and advanced solid tumors.

**Table 1:** Safety and efficacy of HIV positive patients treated with immune checkpoint inhibitors

Study	No of patients with HIV	Disease	Viral response	Disease response
Rai et al. 2017 [20]	11	8 Melanoma, 1 HCC, 1 RCC, 1 BC	No pt. had loss in viral control or immune reconstitution inflammatory syndrome	2 CR, 1 PR, 4 SD, 4PD
Heppt et al. 2017 [19]	10	Melanoma	No increase in viral load, no effect on CD4 count	
Wightman et al. 2015 [18]	1	Melanoma	Improved CD4 count and decrease in Viral load	NA
Burke et al. 2011 [9]	1	Melanoma	Stable CD 4 count and viral load	PR
Morris et al. 2017 [14]	2	Anal cancer	Stable CD4 count and viral load	NA
Sandoval-Sus et al. 2017 [16]	1	Hodgkin lymphoma	Stable CD4 count and viral load	PR
Tomsitz et al. 2017 [17]	1	Melanoma	Stable CD4 count and viral load	PR
Ruzevick et al. 2013 [15]	1	Melanoma	Stable CD4 count and viral load	PR
Davar et al. 2015 [10]	1	Melanoma	Stable CD4 count and viral load	PD
Le Garff et al. 2017 [12]	1	NSCLC	CD4 count increased with stable viral load	PD
Guihot et al. 2017 [6]	1	NSCLC	Stable CD4 count and viral load	PR
Samri et al. 2017 [7]	12	NSCLC	Stable CD4 count and viral load	3 PR, 4 SD, 5 PD
Hentrich et al. 2017 [11]	1	NSCLC	CD4 count decreased but viral load remained stable	PD
McCullar et al. 2017 [13]	1	NSCLC	CD4 count decreased but viral load remained stable	CR
Uldrick et al. 2017 (NCT02595866)	17	Mix	Increase in CD4 count, viral load remained suppressed	NA

Abbreviations: *HCC* hepatocellular carcinoma, *RCC* renal cell carcinoma; *BC* bladder carcinoma, *CR* complete response, *PR* partial response, *SD* stable disease, *PD* progressive disease, *NSCLC* non-small cell lung cancer

In summary, it appears that HIV infection is not an absolute contraindication to CPI therapy. Although the safety is not fully defined, we present a patient who had an exceptional response to therapy with a manageable safety profile. Apart from the therapeutic effect against the malignancy, there are emerging data that the HIV disease itself may be favorably affected by CPI therapy and studies for this indication are also ongoing. Our case and others provide justification for ongoing trials of CPI therapy in patients with HIV infection, a group that has traditionally been excluded from clinical trials.
